# Proteome of a Moraxella catarrhalis Strain under Iron-Restricted Conditions

**DOI:** 10.1128/MRA.00064-20

**Published:** 2020-03-19

**Authors:** Luke V. Blakeway, Aimee Tan, Ian R. Peak, John M. Atack, Kate L. Seib

**Affiliations:** a Institute for Glycomics, Griffith University, Gold Coast, Queensland, Australia; b School of Medical Science, Griffith University, Gold Coast, Queensland, Australia; Indiana University, Bloomington

## Abstract

Moraxella catarrhalis is a leading cause of otitis media and exacerbations of chronic obstructive pulmonary disease; however, its response to iron starvation during infection is not completely understood. Here, we announce a sequential window acquisition of all theoretical fragment ion spectra mass spectrometry (SWATH-MS) data set describing the differential expression of the M. catarrhalis CCRI-195ME proteome under iron-restricted versus iron-replete conditions.

## ANNOUNCEMENT

Moraxella catarrhalis is a human-restricted bacterial pathogen that causes otitis media in children and exacerbations of chronic obstructive pulmonary disease in adults. Iron is a cofactor required by proteins involved in respiration and DNA replication and thus is essential to life for most bacterial species (1). As iron is sequestered by host tissues, all host-adapted pathogens encounter periods of iron starvation. Previous studies have defined the repertoire of genes required for the growth of M. catarrhalis under iron-restricted conditions (2) and described differential expression at the transcriptional level (3). However, global changes in protein expression in response to iron starvation have not been investigated.

M. catarrhalis CCRI-195ME grown in brain heart infusion (BHI) broth with the iron chelator desferal (30 μM) was compared with bacteria grown in BHI only. Cells were grown at 37°C with six replicates per condition. Cell lysates were equalized to 1 μg/μl protein in 50 mM triethylammonium hydrogen carbonate buffer (TEAB). A total of 100 μg of each sample was reduced with 5 mM dithiothreitol at 65°C for 30 mins, alkylated with 10 mM iodoacetamide at 25°C for 30 mins, and digested with 1:25 trypsin at 37°C for 16 hours. Samples were acidified with 1 μl 100% formic acid, pelleted, and resuspended in 100 μl of loading buffer (2% acetonitrile and 0.1% formic acid). Information-dependent acquisition (IDA) and sequential window acquisition of all theoretical fragment ion spectra mass spectrometry (SWATH-MS) experiments were performed by the Australian Proteome Analysis Facility (Sydney, NSW, Australia) using a 5600 TripleTOF mass spectrometer (AB Sciex) with an Ultra nano-liquid chromatography (nanoLC) system (Eksigent) as previously described (4). For 1D IDA, replicates were pooled and 10 μl was injected. For 2D IDA, all samples were pooled and fractionated with high-pH reversed-phase high-performance liquid chromatography (HPLC). A total of 13 fractions were collected, dried, and resuspended in 20 μl of loading buffer, and 10 μl was injected. Protein identification was performed with ProteinPilot (v4.2) in “thorough mode” using the database M. catarrhalis CCRI-195ME coding DNA sequence (CDS) (2,136 proteins) ASM208012v1. SWATH library construction was performed by merging 1D-IDA and 2D-IDA runs using SwathXtend (5) (https://www.bioconductor.org/packages/release/bioc/html/SwathXtend.html). For SWATH-MS, each sample was diluted 1:1 in loading buffer, and 10 μl was injected in random order with one blank run between samples. SWATH data were extracted using PeakView (v2.1) with the following parameters: the 6 most intense fragments of each peptide were extracted (75-ppm mass tolerance and 10-min retention time window), shared and modified peptides were excluded, and peptides with a confidence level of ≥99% and false discovery rate (FDR) of ≤1% were used for quantitation. Peptide peak areas were normalized to the total peak area for each run, and statistical analysis was performed as previously described (4). Proteins are considered differentially abundant if the quantitation ratios were ≥1.5 or ≤0.67 and respective *P* values were ≤0.05.

SWATH-MS identified 695 proteins, of which 94 were differentially abundant under iron-restricted versus iron-replete conditions. Under iron restriction, 39 proteins were more abundant, including the lactoferrin- and transferrin-binding proteins LbpA, LbpB, and TbpB (6, 7), while 55 proteins were less abundant, including the superoxide dismutase SodA (8) and the nitrate reductase subunit NarJ. This data set will act as the primary proteomic resource for studying the response to iron restriction in M. catarrhalis and facilitate study of this important human otopathogen.

### Data availability.

The SWATH mass spectrometry proteomics data and processed differential abundance analysis have been deposited in the ProteomeXchange Consortium via the PRIDE (9) partner repository with the data set identifier PXD017206.
